# A single-center, open label, randomized, controlled study of hydroxychloroquine sulfate in the treatment of low risk PLA_2_R-associated membranous nephropathy

**DOI:** 10.1186/s12882-024-03670-3

**Published:** 2024-07-19

**Authors:** Mei Mei, Jun Zeng, Zhengyang Liu, Li Gong, Li Fang, Quan Hu, Shaofen Huang, Liyin Chai, Xinqing Chen, Haili Sun, Sha Xiang, Chaolin Wen, Bingbing Shen

**Affiliations:** 1 Department of Nephrology & Rheumatology, People’s Hospital of Shapingba District, Chongqing University Shapingba Hospital, School of Medicine，Chongqing University, Chongqing, China; 2 Department of Nephrology, The First Hospital Affiliated to Army Medical University, Chongqing, China; 3 Department of Nephrology, Chongqing Emergency Medical Center, Chongqing University Central Hospital, School of Medicine,Chongqing University, Chongqing, China

**Keywords:** Hydroxychloroquine sulfate, Low risk, PLA_2_R-associated membranous nephropathy

## Abstract

**Objective:**

To evaluate the efficacy and safety of hydroxychloroquine sulfate (HCQ) in the treatment of low risk phospholipase A_2_ receptor (PLA_2_R)-associated membranous nephropathy (MN).

**Methods:**

A total of 110 patients with low risk PLA_2_R-associated MN were included in the study. Patients who met the inclusion and exclusion criteria were assigned randomly to two groups: the HCQ treatment group and the control group. The control group received standard supportive treatment according to the guidelines, while the HCQ treatment group received HCQ in addition to the supportive treatment. The clinical data of the patients were analyzed, with comparisons made at baseline and during the six-month follow-up period. Any adverse reactions were recorded.

**Results:**

The baseline data were comparable between the HCQ treatment group and the control group. At the end of the six-month follow-up period, the reductions in urine protein excretion and serum PLA_2_R antibody titer were more notable in the HCQ treatment group than those in the control group, with these differences being statistically significant (*p* < 0.05). Compared to the control group, the HCQ treatment group had fewer patients who were converted from low risk to moderate-to-high risk (*p* = 0.084). There were also no severe adverse reactions in the HCQ treatment group.

**Conclusion:**

In patients with low risk PLA_2_R-associated MN, adequate supportive therapy combined with HCQ is superior to supportive therapy alone in controlling proteinuria and reducing serum PLA_2_R antibody titers. Additionally, our study demonstrated that the incidence of adverse reactions did not increase.

**Trial registration:**

This study was registered in the Chinese Clinical Trial Registry (Registration No.: ChiCTR1900021757, Date of registration: 2019-03-08).

## Introduction

Primary membranous nephropathy (pMN) is one of the major causes of nephrotic syndrome and end-stage renal disease, its pathogenesis remains inconclusive. While spontaneous remission may occur in some cases of pMN, the majority of patients experience a prolonged disease course, with approximately 30–40% of individuals at risk of progressing to end-stage renal disease [1]. Hou et al. [2] retrospectively analyzed the changes in the types of glomerular diseases in China, reporting a corrected incidence rate of 23.4% for MN. Notably, the adjusted odds for MN increased 13% annually over the 11-year study period, whereas the proportions of other major glomerulopathies remained stable, which exhibited a rapidly increasing trend of MN.

In recent years, the most prominent research advance on pMN was the discovery of the target antigen-M-type phospholipase A_2_ receptor (PLA_2_R) on the surface of normal podocytes [3]. Although several other novel target antigens have been reported recently, PLA_2_R remains the optimal marker for diagnosing and evaluating the efficacy of pMN [4], being identified in 74–78% of the cases of pMN [5, 6]. Based on research advances on pMN, the Kidney Disease: Improving Global Outcomes (KDIGO) reviewed almost all of the 2021 KDIGO guidelines and reassessed the diagnosis and treatment of pMN [7]. Generally, a “wait and see” strategy is used for patients with low risk pMN. However, clinical practice and outcomes indicated that there is still a possibility for some low risk pMN patients who receiving adequate supportive treatment to convert to moderate-to-high risk, and then require immunosuppressive therapy. Therefore, finding a low toxicity immunotherapy for early-stage low risk pMN patients would be highly beneficial in clinical practice. In recent years, hydroxychloroquine sulfate (HCQ) has been used to treat lupus nephritis and IgA nephropathy [8–11], with the mechanisms of action involving repression of T and B lymphocyte activation by inhibition of the degradation of lysosomal derivatives, suppressing presentation of the major histocompatibility complex (MHC)-II-mediated autoantigen, and blocking Toll-like receptor (TLR) and cGAS-STING signals. In addition, HCQ also has effects on anticoagulation by inhibiting the PLA_2_ pathway, and suppressing B cell-activating factors (BAFFs) [12]. Theoretically, HCQ may exert a blocking effect on multiple aspects of the pathogenesis of PLA_2_R-associated MN. The aim of this randomized controlled study was therefore to investigate the efficacy of HCQ in the treatment of PLA_2_R-associated MN.

## Materials and methods

### Research subjects

This study was approved by the Hospital Ethics Committee (KY201994) and registered in the Chinese Clinical Trial Registry (Registration No.: ChiCTR1900021757). The study defined low-risk PLA_2_R-associated MN as: (a) renal biopsy-proven MN and positive for tissue PLA_2_R and IgG4, (b) serum PLA_2_R antibody titer ≤ 100RU/ml (ELISA), and (c) estimated glomerular filtration rate (eGFR) > 60 mL/min/1.72 m^2^ (CKD-EPI formula), urine protein excretion < 4 g/d, and plasma albumin level > 25 g/L. This low risk PLA2R-MN definition made in this study in 2019 is extremely similar to the definition of the low risk group in the 2021 KDIGO guidelines for pMN, which defines low-risk pMN as normal eGFR, proteinuria < 3.5 g/day, and serum albumin > 30 g/L; or normal eGFR, proteinuria < 3.5 g/day; or a > 50% reduction in proteinuria after 6 months of treatment with ACEi/ARB [13]. 

Based on the criteria above,110 patients diagnosed with low risk PLA_2_R-associated MN by the serum PLA_2_R test and renal biopsy between 2019 and 2021 were included in the study. The age of the patients ranged between 18 and 65 years old. In our study, all patients enrolled were treatment-naïve at the time of inclusion. The exclusion criteria for the study included:

1. Patients aged < 18 years or > 65 years
2. those with an Active infection during follow-up
3. those with Other immune diseases, connective tissue diseases, amyloidosis, or requiring immunosuppressive therapy
4. those Administered with any immunosuppressive agents within 6 months before diagnosis
5. those with complex diseases that might interfere with the study or increase its risks, such as diabetes, malignancies, blood diseases, heart or liver diseases, AIDS, or viral hepatitis.

**Fig. 1:**
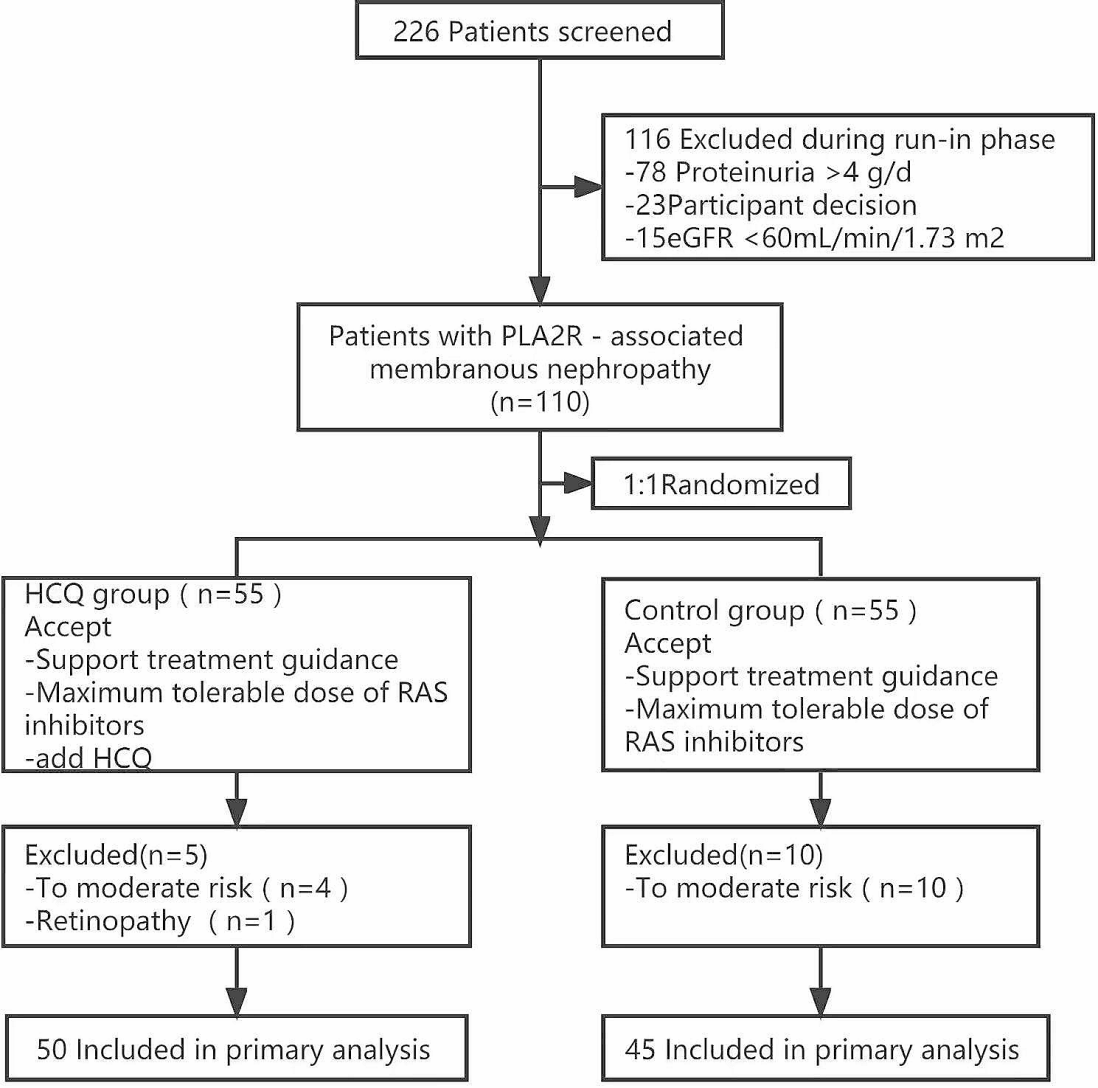
Inclusion flow chart

The details of the criteria for the entire study are presented in Fig. 1.

### Randomization, interventions and withdrawal criteria

All subjects signed written, informed consent and then received a fundus examination prior to the beginning of the research. The eligible subjects were assigned equally 1:1 to the HCQ treatment group or the control group using the computer random number method, with the aim of balancing the treatment groups on potential confounders.

All subjects were administered adequate supportive therapy by trained investigators, including cessation of smoking and drinking, diet management, moderate exercise, and maximum tolerable renin-angiotensin system inhibitors (RASIs). The specific optimization protocol for RASIs is as follows: Initiate treatment at the recommended dose according to the product label. Gradually increase the dose based on monitoring of blood pressure, serum potassium levels, renal function, and other potential adverse effects. If any of these adverse effects occur, reduce the dose accordingly, until the maximum tolerated dose for the patient is reached. Prior to reaching the maximum tolerated dose, weekly monitoring of blood pressure, serum potassium levels, and renal function is required, along with regular follow-up for general health status by a physician. After safely reaching the maximum tolerated dose, the patient will enter the planned regular follow-up schedule.

The investigators also recorded and provided guidance on managing adverse reactions. The HCQ treatment group was given oral HCQ (SPH Zhongxi Pharmaceutical Co., Ltd.) based on adequate supportive therapy in the control group; 400 mg/d for patients ≥ 50 kg and 300 mg/d for those < 50 kg. Our investigators conducted weekly telephone follow-up visits with patients to ensure that they took their medication as prescribed. Patients who developed HCQ-related retinal toxicity (evaluated by an investigator with the assistance of an ophthalmologist) and Qtc prolongation (Electrocardiogram examination once a month)were withdrawn from the study. PLA_2_R antibody titers were re-examined at the 3rd and 6th months of the follow-up period, the levels of urine protein, plasma albumin, serum creatinine, coagulation function, and other relevant indicators were re-examined every month.

Moderate-to-high risk pMN in the study was considered in subjects who met one of the following conditions at the 3rd month of follow-up and due to the requirement for additional immunosuppressive therapy were withdrawn from the study: (1) increase in urine protein excretion of > 50% compared to baseline or > 6 g/24 h, (2) increase in serum creatinine level by > 30% compared to baseline, and (3) an increase in PLA_2_R antibody titer by > 50% compared to baseline and an increase in the levels of urine protein or serum creatinine over those measured at baseline.

### Study outcomes

The percentage change in the quantity of 24-hour urine protein excretion from baseline to the 6th month of follow-up was selected as the primary endpoint. A reduction of 50% or above in 24-hour urine protein excretion was considered to represent effective treatment.

The secondary endpoints included the percentage changes in serum PLA_2_R antibody titer, eGFR and plasma albumin level, and the number of patients converted to moderate-to-high risk. The adverse reactions were also recorded.

### Statistical analysis

Data with a normal distribution were expressed as mean ± standard deviation, while non-normally distributed data were expressed as medians and interquartile range (Q_25 −_ Q_75_). Categorical data were expressed as counts and percentages. The baseline characteristics were compared between the two groups using the paired-sample *t*-test, Wilcoxon rank-sum test (continuous variables) or χ^2^ test (nominal variables). Univariate and multivariate logistic regression analyses were also used to determine the independent predictors for at least a 50% reduction in urine protein excretion. A *p*-value < 0.05 was considered statistically significant. SPSS 19.0 and SAS 9.4 software were used for the statistical analyses.

## Results

### Baseline features

A total of 110 PLA_2_R-associated MN patients who met the inclusion criteria were selected from October 2019 to October 2021 and categorized randomly into the HCQ treatment group and the control group. 5 patients in the treatment group and 10 patients in the control group withdrew from the study respectively. In the HCQ treatment group, 4 patients withdrew due to progression to moderate risk, and 1 patient withdrew due to retinal toxicity. In the control group, 10 patients withdrew due to progression to moderate risk (Fig. 1). No significant differences were observed in the baseline clinical features between the two groups (*p* > 0.05) (Table 1).

**Table 1:** Baseline features of the two groups of patients

Baseline variable	HCQ treatment group (*n* = 50)	Control group (*n* = 45)	Statistic	*p*
Age (yr)	51.5 ± 12.2	50.9 ± 13.1	0.23	0.8177
Male ratio	28 (56.0%)	32 (71.1%)	2.32	0.1274
Mean arterial pressure (MAP, mmHg)	101.2 ± 10.2	102.9 ± 8.4	0.88	0.3816
Serum creatinine (Scr, µmol/L)	117.0 ± 36.9	121.0 ± 38.1	0.52	0.6047
eGFR (mL/min/1.73 m^2^)	80.7 ± 12.5	79.2 ± 7.2	0.72	0.4753
24-hour urine protein quantity (g/24 h)	3.38 (2.55, 3.74)	3.23 (2.63, 3.88)	0.11	0.9078
Serum PLA_2_R antibody titers (RU/ml)	60.0 (40.0, 80.0)	60.0 (30.0, 64.0)	0.73	0.4657
BMI	24.8 ± 2.9	25.0 ± 3.0	0.23	0.8219
Plasma albumin (g/L)	29.8 (27.5, 32.5)	29.1 (27.1, 32.2)	0.47	0.6359
Use of RASI				
Maximum tolerable ARB	20 (40.0%)	22 (48.9%)	0.76	0.3837
Maximum tolerable ACEI	10 (20.0%)	8 (17.8%)	0.08	0.7826

No significant differences were identified in the baseline features between the two groups (*p* > 0.05)

### Maximum tolerable RASIs

We separately counted the number of patients in the control group and the HCQ group who exceeded the maximum recommended dose of RASIs according to the product label and those who did not reach the maximum recommended dose. Statistical analysis was conducted. The results showed that in the control group, 10 patients (22.2%) exceeded the maximum recommended dose of RASIs, and 4 patients (8.9%) did not reach the maximum recommended dose. In the HCQ group, 12 patients (24%) exceeded the maximum recommended dose of RASIs, and 4 patients (8%) did not reach the maximum recommended dose. There was no statistically significant difference between the two groups in the use of the maximum tolerated dose of RAS inhibitors (Table 2).

**Table 2:** Primary and secondary endpoints

Indicator	HCQ treatment group (*n* = 50)	Control group (*n* = 45)	Statistic	*p*
**Primary endpoint**				
Percentage change in 24-hour urine protein excretion at the 3rd month	-25.9% (13.0%, 46.9%)	-9.4% (0.7%, 36.4%)	1.76	0.0779
Percentage change in 24-hour urine protein excretion at the 6th month	-50.2% (28.4%, 65.6%)	-28.2% (1.6%, 50.0%)	2.93	0.0034
24-hour urine protein excretion at the 3rd month (g/24 h)	2.44(1.35,2.85)	2.98(1.47,2.97)	0.540	0.185
24-hour urine protein excretion at the 6th month (g/24 h)	1.75(0.98,2.05)	2.67(1.27,2.)	6.396	0.001
**Secondary endpoint**				
PLA_2_R				
Percentage change in PLA_2_R antibody titers at the 3rd month	-21.9% (0%, 33.3%)	-1.0% (0%, 25.0%)	2.32	0.0203
Percentage change in PLA_2_R antibody titers at the 6th month	-50.0% (20.0%, 62.5%)	-25.0% (0%, 50.0%)	2.60	0.0092
PLA_2_R antibody titers at the 3rd month (u/ml)	41.8 (31.2, 56.3)	47.4 (32.6, 58.5)	1.817	0.069
PLA_2_R antibody titers at the 6th month (u/ml)	34.6 (28.4, 42.8)	41.3 (30.3, 54.2)	2.837	0.017
eGFR				
Percentage change in eGFR at the 3rd month	0% (-1.6%, 1.6%)	0.3% (-1.2%, 1.5%)	0.68	0.4952
Percentage change in eGFR at the 6th month	-0.7% (-2.3%, 0.7%)	-0.7% (-2.7%, 0.2%)	0.26	0.7971
eGFR at the 3rd month	80.4 ± 12.9	79.1 ± 13.8	0.474	0.636
eGFR at the 6th month	81.6 ± 13.4	80.2 ± 14.1	0.496	0.621
Albumin				
Percentage change in albumin at the 3rd month	8.5% (4.5%, 18.8%)	8.4% (5.3%, 17.6%)	1.217	0.224
Percentage change in albumin at the 6th month	15.4% (7.7%, 23.6%)	11.0% (-8.4%, 4.5%)	4.283	0.0001
albumin at the 3rd month	32.3 (29.5, 36.4)	31.7 (29.1, 35.5)	0.487	0.626
albumin at the 6th month	34.4 (31.2, 38.3)	32.3 (29.6, 35.9)	1.703	0.089
ACEi/ARB dose				
Number of patients who reduced manual recommended ACEi/ARB dose	12(24%)	10(22.2%)	0.205	0.837
Number of patients who increased manual recommendedACEi/ARB dose	4(8%)	4(8.9%)	0.156	0.876

The percentage changes in 24-hour urine protein excretion, PLA_2_R antibody titers, and albumin at the 6th month of follow-up were statistically different (*p* < 0.05), while that of eGFR showed no statistical difference (*p* > 0.05)

### Primary endpoints: percentage change in 24-hour urine protein excretion

The 24-hour urine protein excretion was roughly similar at baseline in the HCQ treatment group and the control group, with no statistical difference observed (*p* = 0.9198). Both groups of patients showed a decrease in proteinuria, with the treatment group showed a more significant decrease. At the 6th month of follow-up, both groups of patients had a decrease in proteinuria compared to baseline (1.75 g vs. 2.67 g) (Table 2). At the 3rd month of follow-up, excretion had declined relative to baseline levels in both groups (25.9% vs. 9.4%), with the difference being similar but not statistically significant (*p* = 0.0779). At the 6th month of follow-up, the HCQ treatment group showed a more notable reduction in 24-hour urine protein excretion than that in the control group (50.2% vs. 28.2%), with this difference being statistically significant (*p* = 0.0034) (Table 2).

### Secondary endpoints

#### Percentage Change in PLA_2_R antibody titers

The PLA_2_R antibody titers were similar at baseline between the HCQ treatment group and the control group, with no statistically significant differences. (*p* = 0.4657). At the 3rd month of follow-up, the HCQ treatment group showed statistically significant lower PLA_2_R antibody titers compared with those in the control group (-21.9% vs.-1.0%; *p* = 0.0203). At the 6th month of follow-up, the difference in the change of PLA2R antibody titers was also statistically significant between the HCQ treatment and control group (-50.0% vs. -25.0%, *p* = 0.00092) (Table 2).

#### Change in eGFR

There was no statistically significant difference in eGFR at baseline between the HCQ treatment group and the control group (*p* = 0.4753). The eGFR of both groups of patients remained stable during follow-up. At the 3rd and 6th months of follow-up, the change in eGFR was also not significantly different between the two groups (*p* = 0.4952, 0.7971) (Table 2).

#### Change in serum albumin levels

Serum albumin levels showed no significant difference at baseline between the HCQ treatment group and the control group (*p* = 0.6359). At the 3rd month of follow-up, the HCQ treatment group had no noticeable increase in serum albumin level than that observed in the control group (8.5% vs. 8.4%). At the 6th month of follow-up, the increase in albumin level was significantly greater in the HCQ treatment group than that of the control group (15.4% vs. 11.0%, *p* = 0.0001) (Table 2).

### Comparison of treatment responses at 6 months

At the 6th month of follow-up, there were 15 cases in the HCQ treatment group where PLA2R antibody titers decreased by more than 50%, which was higher than the control group (7 cases). This difference was not statistically significant (*p* = 0.0956). At the 6th month of follow-up, the number of patients with a > 50% decline in 24-hour urine protein excretion was 26 cases in the HCQ treatment group and 12 cases in the control group, with this difference being statistically significant (*p* = 0.0118) (Table 3).

**Table 3:** Comparison of the number of patients with reductions of more than 50% in PLA_2_R antibody titers and 24-hour urine protein excretion at the 6th month of follow-up between the two groups

Variable	HCQ treatment group (*n* = 50)	Control group (*n* = 45)	χ^2^	*p*
More than 50% reduction in PLA_2_R antibody titers	15 (30.0%)	7 (15.6%)	2.78	0.0956
More than 50% reduction in 24-hour urine protein excretion	26 (52.0%)	12 (26.7%)	6.33	0.0118

At the 6th month of follow-up, there was a statistically significant difference in the number of patients with more than a 50% reduction in 24-hour urine protein excretion between the two groups. The number of patients with more than a 50% reduction in PLA_2_R antibody titers was similar in the two groups, with no statistically significant difference observed

### Number of patients with conversion to moderate-to-high risk

After the completion of the 6-month follow-up, 5 patients in the HCQ treatment group withdrew from the study, including 4 patients with conversion to moderate-to-high risk due to an increase in urine protein excretion of > 50% relative to baseline level and greater than 6 g/24 h. In the control group, 10 patients with conversion to moderate-to-high risk withdrew from the study, including 3 patients with an increase in serum creatinine level of > 30% relative to baseline level, and 7 patients with an increase in urine protein excretion of > 50% relative to baseline and greater than 6 g/24 h. Therefore, the probability of conversion from low risk to moderate-to-high risk was smaller in the HCQ treatment group than in the control group (4 vs. 10), close but not statistically significant (*p* = 0.086) (Table 4).

**Table 4:** Comparison of the number of patients with conversion to moderate-to-high risk between the two groups

	HCQ treatment group	Control group	*p*
Number of patients at baseline	55	55	
Number of patients with conversion to Moderate-to-high risk	4 (7.3%)	10 (18.2%)	0.086

The difference in the number of patients with conversion to Moderate-to-high risk between the two groups was not statistically significant (*p* = 0.086)

### Independent predictors analysis

We used univariate and multivariate logistic regression to analyze independent predictors affecting the reduction rate of effective albuminuria (by at least 50%). The results showed that in univariate analysis, HCQ was the only independent predictor of reduction in proteinuria by > 50%, see Table 5.

**Table 5:** Independent predictors of reduction in proteinuria by > 50%univariate and multivariate logistic regression analysis

Characteristics	Univariate analysis			Multivariate analysis		
	OR	95% CI	*P*values	OR	95% CI	*P*values
Age	1.0	(0.95–1.03)	0.78	1.01	(0.96–1.04)	0.77
Gender	1.14	(0.82–1.44)	0.57	1.12	(0.85–1.37)	0.68
eGFR at baseline	1.02	(0.98–1.05)	0.62	1.0	(0.97–1.03)	0.79
proteinuria at baseline	0.84	(0.69–1.01)	0.15	0.79	(0.69–1.01)	0.09
serum PLA2R antibody titers at baseline	0.86	(0.67–0.97)	0.17	0.73	(0.61–0.88)	**0.045**
HCQ	1.76	(1.37–2.14)	**0.029**	2.15	(1.67–3.01)	**0.005**

Statistically significant *P* values are shown in bold

### Adverse reactions

The difference in adverse reactions was not significantly different between the two groups, with no severe adverse reactions occurring in either group (Table 6). In the HCQ treatment group, one patient who presented with blurred vision withdrew from the study due to suspected HCQ-related retinal toxicity. No significant changes in QT intervals were observed in either the treatment HCQ group or the control group during the study period. In addition, there were 3 patients with skin pruritus, 2 patients with nausea, hepatic dysfunction, skin rashes and palpitation, and 1 patient with abdominal pain and dizziness, all of which were relieved after symptomatic treatment. In the control group, 2 patients had nausea, skin pruritus, and skin rashes and 1 patient each had either hepatic dysfunction, abdominal pain, palpitation, or dizziness. The evaluation in combination with six months of medication indicated that the safety of drugs was good in the two groups.

**Table 6:** Adverse reactions in the two groups

Adverse reaction	Number of cases	
	HCQ treatment group	Control group
Severe adverse reactions	0	0
Adverse reactions		
Number of patients without adverse reactions	32	32
Number of patients with one adverse reaction	8	6
Number of patients with more than 2 adverse reactions	2	2
Types of adverse reactions		
Nausea	2	2
Abdominal pain	1	1
Hepatic dysfunction	2	1
Palpitation	2	1
Dizziness	1	1
Skin rashes	2	2
Skin pruritus	3	2
Blurred vision	1	0

## Discussion

Given that the low risk pMN patients defined at the time of inclusion in this study in 2019 satisfied the definition described in the 2021 KDIGO guidelines, the research findings can serve as a reference for clinical practice [13]. This study investigated the efficacy and safety of adequate supportive therapy combined with HCQ in the treatment of patients with low risk PLA_2_R-associated MN, with the results of the 6th month follow-up showing that the HCQ treatment group had a noticeable reduction in 24-hour urine protein excretion (50.2% vs. 28.2%) and PLA_2_R antibody titers (50% vs. 25%) compared to those measured in the control group. The number of patients with a > 50% reduction in PLA_2_R antibody titers was greater in the HCQ treatment group than in the control group (15 vs. 7), although this difference was not statistically significant (*p* = 0.0956). The number of patients with more than 50% decline in 24-hour urine protein excretion was also significantly greater in the HCQ treatment group than that in the control group (26 vs. 12; *p* = 0.0118). Although the number of patients with conversion to moderate-to-high risk was close to but did not reach statistical significance, based on the proteinuria reduction and immunomodulatory mechanisms of hydroxychloroquine sulfate, we speculate that this is related to an insufficient sample size. Therefore, further research with an expanded sample size is both feasible and necessary. We speculate from these findings that HCQ reduces PLA_2_R antibody titers and proteinuria and also lowers the probability of conversion from low risk to moderate-to-high risk by immunomodulatory function. This indicates that HCQ has potential as a therapeutic agent. Our data also shows that the HCQ group was comparable to the control group in terms of the incidence rate of adverse reactions. In the present study, nausea, abdominal pain, hepatic dysfunction and skin pruritus were the main adverse reactions, similar to those reported in previous studies. However, these reactions had no obvious impacts on the patients’ life, with all the reactions being relieved after symptomatic treatment. In particular, retinal toxicity, which is of more concern [14], led to blurred vision in only one patient who subsequently withdrew from the study.

The high heterogeneity of clinical outcomes is viewed as one of the toughest problems in the treatment of pMN. Currently, spontaneous remission occurs in about one-third of pMN patients, although it is very difficult to precisely evaluate whose symptoms can be relieved spontaneously and when they will be relieved, with the optimal clinical protocol remaining inconclusive [15–17]. The newly issued KDIGO guidelines classify pMN patients into three risk groups, namely, low risk group, moderate risk group, and high risk group, with different therapies recommended in these three groups. For instance, a “wait and see” strategy is proposed for patients with low risk pMN, based on adequate supportive therapy [18]. However, in clinical practice, disease progression may also appear in low risk pMN patients who have received adequate supportive treatment and immunosuppressive therapy has to be initiated. Practical evidence has confirmed that both spontaneous remission and post-treatment remission contribute to the long-term prognosis of pMN patients [19]. As such, low-toxicity and effective immunomodulatory therapy that can help low risk patients achieve a favorable immune response and remission at an early stage is of great clinical significance.

HCQ possesses pharmacological effects such as immunomodulation, anti-inflammation and anti-thrombosis and has been proven to be effective in treating rheumatoid arthritis, systemic lupus erythematosus, and Sjogren’s syndrome by protecting target organs [20–23]. In recent years, HCQ has also been shown to relieve proteinuria in patients with IgA nephropathy [24, 25]. Additionally, HCQ may have direct effects on podocyte function and integrity, potentially stabilizing the podocyte cytoskeleton and reducing podocyte injury and protein leakage into the urine [26]. In PLA2R-associated membranous nephropathy, the risk allele of human leukocyte antigen-D locus is expressed on antigen-presenting cells and can present the epitope PLA2R to activate CD4 + helper T cells, and then drive the differentiation of activated B cells into plasma cells to produce anti PLA2R antibodies [27]. HCQ increases regulatory T cell activity and several pro-inflammatory cytokines by downregulating the co stimulatory molecule CD154 on CD4 + T. HCQ can also interfere with lysosomal activity and autophagy by increasing the pH of endosomal compartments, thereby inhibiting the expression of major histocompatibility complex (MHC) II molecules and antigen presentation [12]. Analysis from a theoretical perspective indicates that HCQ possibly reduces the generation of pMN-specific antibodies by suppressing antigen presentation and lymphocyte activation, and it is also conducive to the treatment of pMN by protecting the kidney, inhibiting thrombosis and reducing proteinuria. Moreover, it is worth noting that HCQ has a favorable safety profile during normal use, a necessary requirement for immunotherapy in patients with low risk pMN [28].

Given that the low risk pMN patients defined at the time of inclusion in this study in 2019 satisfied the definition described in the 2021 KDIGO guidelines, the research findings can serve as a reference for clinical practice [13]. This study investigated the efficacy and safety of adequate supportive therapy combined with HCQ in the treatment of patients with low risk PLA_2_R-associated MN, with the results of the 6th month follow-up showing that the HCQ treatment group had a noticeable reduction in 24-hour urine protein excretion (50.2% vs. 28.2%) and PLA_2_R antibody titers (50% vs. 25%) compared to those measured in the control group. The number of patients with a > 50% reduction in PLA_2_R antibody titers was greater in the HCQ treatment group than in the control group (15 vs. 7), although this difference was not statistically significant (*p* = 0.0956). The number of patients with more than 50% decline in 24-hour urine protein excretion was also significantly greater in the HCQ treatment group than that in the control group (26 vs. 12; *p* = 0.0118). We speculate from these findings that HCQ reduces PLA_2_R antibody titers and proteinuria and also lowers the probability of conversion from low risk to moderate-to-high risk by immunomodulatory function. This indicates that HCQ has potential as a therapeutic agent. Our data also shows that the HCQ group was comparable to the control group in terms of the incidence rate of adverse reactions. In the present study, nausea, abdominal pain, hepatic dysfunction and skin pruritus were the main adverse reactions, similar to those reported in previous studies. However, these reactions had no obvious impacts on the patients’ life, with all the reactions being relieved after symptomatic treatment. In particular, retinal toxicity, which is of more concern [14], led to blurred vision in only one patient who subsequently withdrew from the study. However, the study was limited in that it was an open label, single-center study with a small sample size and short treatment course and follow-up period, and therefore the long-term efficacy of HCQ in the treatment of patients with low risk pMN could not be evaluated. Although this study was an early-phase exploratory trial, the potentially effective approach for treating low risk pMN patients it demonstrated should not be ignored. However, the efficacy of HCQ needs to be validated by a large-scale, long-term, multicenter clinical trial. Furthermore, none of the patients in our study received SGLT2 inhibitors. These limitations underscore the need for future studies to explore the comparative effectiveness of HCQ in combination with SGLT2 inhibitors, as well as to investigate the potential synergistic effects of these therapies in managing proteinuria CKD.

## Limitations

This study has several limitations that should be acknowledged. The sample size was relatively small and the study was unblinded. Additionally, the follow-up duration may not have been sufficient to capture long-term effects of HCQ treatment. Spontaneous remission is recognized as a potential confounder in studies of low-risk MN, with KDIGO guidelines highlighting its occurrence in a substantial proportion of patients. While our study design included assessments at 3 and 6 months to evaluate the efficacy of HCQ, the possibility of spontaneous remission influencing our observed outcomes cannot be fully excluded. To address these limitations, we recommend the design of a larger, multicenter, randomized controlled trial (RCT) to validate our findings. Such a trial should include blinding of participants and investigators to reduce bias.

## Conclusion

Data from this preliminary study confirmed that adequate supportive therapy combined with HCQ reduces PLA_2_R antibody titers and proteinuria. We consider that HCQ may be a potential add-on therapy for patients with low-risk MN during the first 6 months after diagnosis, in addition to the traditional maximal anti-proteinuria therapy, which is worthy of further investigation. Additionally, the findings of this study warrant validation in larger multicenter trials.

## Data Availability

All data generated or analysed during this study are included in this published article.
